# *Catharanthus roseus* Combined with Ursolic Acid Attenuates Streptozotocin-Induced Diabetes through Insulin Secretion and Glycogen Storage

**DOI:** 10.1155/2020/8565760

**Published:** 2020-02-18

**Authors:** Huda Mohammed Alkreathy, Aftab Ahmad

**Affiliations:** ^1^Department of Pharmacology, Faculty of Medicine, King Abdulaziz University, Jeddah, Saudi Arabia; ^2^Health Information Technology Department, Faculty of Applied Studies, King Abdulaziz University, Jeddah -21589, Saudi Arabia

## Abstract

*Catharanthus roseus* (*C. roseus*) and ursolic acid (UA) are ayurvedic medicines with multiple pharmacological activities including antidiabetic activity, but till date, no study is available on their combination. This study documented the antidiabetic efficacy of the combination of *C. roseus* and UA in rats. Rats were divided into six groups. All groups were given a single dose of Streptozotocin (STZ) at a dose of 50 mg/kg by intraperitoneal route for induction of diabetes, except the normal control group. Group 1 was treated as a normal control (NC) group and fed with saline water, Group 2 as a Diabetes Control group, Group 3 as a STZ+*C. roseus* ethanolic extract (CREE) group at 50 mg/kg p.o., Group 4 as a STZ+UA group orally at 50 mg/kg, Group 5 as a STZ+CREE (25 mg/kg p.o.)+UA (25 mg/kg p.o.) group, and Group 6 as a STZ+Glimepiride (0.1 mg/kg) group. Diabetes was confirmed after 72 hours by estimation of blood glucose level, and then treatment was given for the next 28 days. During the course of treatment, plasma insulin and blood glucose were measured regularly at the interval of 7 days. At the end of the protocol, blood was collected and animals were sacrificed. The glucose level, insulin level, liver glycogen storage level, and antioxidant enzymes (LPO, CAT, SOD, GPx, GST) were measured. The blood glucose level in Group 5 significantly (*P* < 0.001) reduced to 98.35 ± 2.45 mg/dl in comparison with that in Group 2 (321.75 ± 5.46 mg/dl). The level of plasma insulin in Group 5 increased (13.65 ± 0.10 *μ*U/ml) significantly (*P* < 0.01) as compared with that in Group 2 (05.93 ± 0.31 *μ*U/ml). In Group 5, the level of glycogen in liver was significantly (*P* < 0.01) increased as compared with that in Group 2 rats. The level of antioxidant enzymes in Group 5 restored toward normal values significantly (*P* < 0.01; *P* < 0.001) as compared with that in Group 2 animals. These findings suggest that low-dose combination of CREE and UA is effective in the treatment of diabetes.

## 1. Introduction

Diabetes is a metabolic disease which induced either due to the inability of pancreas to secrete sufficient insulin in body or when body is unable to use insulin effectively for the regulation of blood sugar. In both conditions, a large amount of sugar remains in blood. The increased sugar of blood is an identifying parameter for diabetes and this increased level for long time creates severe injury to multiple organ systems of the body. In year 2014, 8.5% of the adults suffered from diabetes. In year 2016, 1.6 million casualties were linked with diabetes. According to the 2011 Diabetes National Fact Sheet, around 8.3% people in the United States suffer from diabetes. Around 27% of the people already suffering from diabetes do not have knowledge that they have diabetes. The number of patient of diabetes increased more than 400% from year 1980 to 2014. Prevalence of diabetes increased mainly in undeveloped and developing countries, where income of the peoples is low. The diabetes is a main causative factor of kidney failure, blindness, stroke, heart attacks, and the amputation of limb. In year 2016, around 1.60 million casualties were associated with diabetes. Further, 2.20 million casualties were associated with the increased sugar level of blood in the year 2012. World Health Organization estimated that diabetes was the 7^th^ major reason of casualty in the year 2016. This is estimated that approximately 425 million adults were suffering from diabetes in the year 2017 around the globe, as per the reports of the International Diabetes Federation. This is further postulated that the total prevalence of diabetic patients is expected to increase up to 629 million by the year 2045 [1]. Lifestyle management is an essential feature of diabetes care, which includes physical work, exercise, healthy food, psychosocial care, maintaining normal weight of body, and avoiding the use of tobacco can delay or prevent the induction of type 2 diabetes mellitus [2].

*Catharanthus roseus* (*C. roseus*), popular with periwinkle synonym, is a flowering plant that belongs to the Apocynaceae family. In the ayurvedic system of medicine, its root and bark extract are used traditionally for the treatment of various kinds of diseases. In the Chinese alternative medicine system, decoction was utilized for the treatment of various disorders, which includes Hodgkin lymphoma, malaria, as well as diabetes. Previously, various alkaloids were extracted from this plant, of which vincristine and vinblastine got significant importance and utilized for the management of Hodgkin lymphoma and blood cancer [3].

Ursolic acid (UA) is a very unique triterpenoid molecule present in medicinal plant, kingdom plantae, and utilized as an important part of the human food. Various recently studies reported the wide spectrum pharmacological potentials of UA. It exhibits a variety of effects including antimicrobial, anti-arthritic, and anticancer effects against variety of cancers [4, 5]. Numerous bioactivities of UA have been reported, but till date no clinical work available which demonstrates beneficial effects to human health care. In pre-clinical study, UA decreases the growth of many cancerous cells by inhibition of STAT-3 pathway in cell [6, 7] and also reduce cancerous cell proliferation by induction of apoptotic pathway [8, 9]. UA also inhibits JNK-expression and activation of IL-2 of JURKAT leukemia T-Cells which leads to the proliferation reduction and activation of T-Cell [10]. As per previous work, UA is a weak inhibitor of aromatase enzyme [11], and it increases the brown fat and muscle amount and decreases the obesity of white fat and related conditions when present in fed of mice. UA under physiological concentrations also induces erythrocyte apoptosis (programmed cellular death of red blood cells). It also reduces the atrophy of muscle and enhances the growth of muscles in mice, which also exhibits significant cardio-protection activity [12]. In animals, UA shows neuronal regeneration property after injury of nerve. UA is effective in the treatment of cognitive impairment in animals. UA is also used in the improvement of cognitive disorders by blockage of stress of endoplasm reticulum and nuclear factor-*κ*B/I*κ*B kinase *β*-mediated pro-inflammatory path in animals. UA improves lipopolysaccharide-induced cognition disorder in brain of mice by suppression of p38/NF-*κ*B-inflammatory path. UA improves cognition impairment and modulates oxidative stress damage in mice brain induced by D galactose. UA improves regeneration of liver of mice after partial removal of part of liver [13]. UA modulates cellular immunity and improve function of pancreas *β*-cell in diabetic high fat diet animals. It increases mass of skeletal muscle, exercise capacity, and griping strength. UA attenuates aging metabolic-phenotype by promotion of rejuvenation of skeletal muscle [9]. Recently, a pharmacological and docking studies on the influence of UA on adjuvant-induced arthritis reported that *Ocimum sanctum* L leaf extract (rich in UA) loaded nanostructured lipid carriers inhibited the COX-1, COX-2, IL-1, and TNF-*α*; hence, ameliorate the arthritis in rats [14]. Moreover, the *in vitro* anti-proliferative activities of some novel synthetic quinoline derivatives of UA bearing hydrazide, oxadiazole, or thiadiazole moieties against three cancer cell lines (MDA-MB-231, HeLa, and SMMC-7721) have been recently reported [15]. *C. roseus* and UA are ayurvedic medicines which possess numerous pharmacological activities including antidiabetic activity, but till date, no study is available on their combination against STZ-induced diabetes in rats. Based on the published literature, it was thought worthwhile to evaluate the antidiabetic activity of *C. roseus* ethanolic extract (CREE) alone, UA alone, and combination of both *C. roseus* ethanolic extract (CREE) and UA. Further, it gives the idea about the beneficial effect of low-dose combination of *C. roseus* ethanolic extract (CREE) and UA. In view of the above background, the current study was designed for evaluation of antidiabetic potential of combination of *C. roseus* ethanolic extract (CREE) and UA in STZ-induced diabetic rats.

## 2. Materials and Methods

### 2.1. Chemicals and Drugs

Ursolic acid (UA) and Streptozotocin (STZ) were purchased from Sigma-Aldrich Chemical Company, St. Louis, (MO, USA). All other chemicals and solvents used in the study were of analytical grade and procured from an approved vendor. Standard pellet diet for animals was obtained from local supplier.

### 2.2. Ethanolic Extraction of Catharanthus roseus Leaves

Periwinkle leaves (*C. roseus* leaves) were collected from the gardens in the campus of King Abdulaziz University, Jeddah, and was authenticated by one of the botanist at King Abdulaziz University. Leaves were dried in shadow by avoiding direct sunlight for several days. After drying, the leaves were crushed into a coarsely powdered state and 0.1 kg of powdered drug was mixed in petroleum-ether and left aside for two days for removing all chloroplast, wax, and fats. After this, it was filtrated and then suspended in 95% of 500 mL alcohol, and then extracted by using soxhlet apparatus for eighteen hours. After that, ethanol was evaporated by using rotary-evaporator at 40–44°C temperature. After significant evaporation, thick concentrated extract of ethanol was formed which was filtered by the use of a filter paper (coarse sieve). Then obtained filtrate was dried with reduced pressure and lastly it was lyophilized. This dried sample was used for experimentation purpose. The prepared *C. roseus* ethanolic extract (CREE) at a dose of 50 mg/kg of body weight was given orally, based on the previous published reports [16, 17].

### 2.3. Animals

Healthy male Wistar strain rats (5-6 months old) weighing 100-200 g were procured from the animal facility of King Fahad Medical Research Centre, King Abdulaziz University-Jeddah, Saudi Arabia. This research study was carried out by adopting the guidelines of the Institutional Animal Ethical Committee (IAEC) on the use and care of laboratory animals. The experimental rats were arbitrarily segregated into groups in different cages in standard set-controlled conditions. Before the commencement of the dosing, the rats were acclimatized in the animal room for seven days by maintaining a temperature of 25°C ± 2°C; relative humidity of 30-70%; and 12:12 h light/dark cycle. The rats were given free access to standard quality pallet diet and tap water *ad libitum*.

### 2.4. Initiation of Diabetes in Rats

STZ was be used for the induction of diabetes in rats. The animals were in fasting for sixteen hours before the diabetes induction. STZ was freshly prepared in the 0.05 M citrate buffer with value pH 4.5. A single dose of STZ (50 mg per kg, body weight) was administered to the rats by intraperitoneal injection to induce the diabetes [18]. The diabetes induction was accessed by polyuria, polydipsia, and by the measurement of concentrations of blood glucose after 72 hours of administration of Streptozotocin and then treatment was given for next 28 days.

### 2.5. Experimental Design

Animals were acclimatized and allocated randomly in the six groups (*n* = 6) and rats were treated as per the following schedule for the period of 28 days. The body weights of the experimental rats were recorded using an electronic weighing balance before starting the treatments and at the termination of the protocol.

Group 1 (normal control group): the rats were fed with normal saline (0.9%) and served as normal control (NC).

Group 2 (Diabetes Control group): diabetes was induced by a single intraperitoneal injection of STZ (50 mg/kg, body weight).

Group 3 (CREE treated group): STZ+*C. roseus* ethanolic extract (CREE) at a dose of 50 mg/kg of body weight by oral route.

Group 4 (UA treated group): STZ+UA (50 mg per kg, body weight) by oral route.

Group 5 (CREE+UA treated group): STZ+CREE (25 mg per kg of body weight, orally)+UA (25 mg per kg of body weight, orally).

Group 6 (Glimepiride-treated group): STZ+Standard Glimepiride (0.1 mg/kg, orally).

### 2.6. Estimation of Biochemical Parameters

At the end of protocol, the whole blood was withdrawn from the retro-orbital cavity of the animal in the light anesthesia. On the termination, blood was left aside to stand for thirty minutes at room temperature without anticoagulant. Then it was subjected to centrifuge for ten minutes at 2500 rpm to separate the serum at 4°C. The obtained serum was then held back at lower temperature (2-3°C) for further measurement of different biochemical study. Serum insulin and glucose levels were determined by enzymatic methods with the help of an automated analyzer (Dimension Vista® system, Siemens, Germany) using standard biochemical kits [19].

### 2.7. Estimation of Oxidative Stress Markers

The animals were euthanized by chloroform overdose, and their liver was isolated and washed in normal saline solution (0.9% NaCl solution in distilled water). The liver tissues were then perfused with 50 mmol/L sodium phosphate buffer solution (pH 7.4), which also contained EDTA (0.1 mmol/L), to drain the blood clots and cells. The part of the liver was homogenized in phosphate buffer (0.1 M; pH 7.4) and carefully centrifuged at 10,000 rpm, 15 minutes at 4°C. The clear supernatant was then used to assess antioxidant enzymes such as Glutathione peroxidase (GPx), Glutathione-S-transferase (GST), superoxide dismutase (SOD), catalase (CAT), and lipid peroxidation (LPO) by the method of Shivavedi et al., 2017 [20].

### 2.8. Statistical Analysis

All the data was analyzed using the SPSS (Statistical Package for the Social Sciences) version 20.0 software (SPSS Inc., Chicago, IL, USA). The data was expressed as Mean ± SEM. The significance among different groups was determined by using one-way analysis of variance (ANOVA) test. *P* value of ^∗^*P* < .05, ^∗∗^*P* < .01, and ^∗∗∗^*P* < .001 were considered to be statistically significant, when compared with the control group.

## 3. Results

### 3.1. Effect on Body Weight

**Figure 1:**
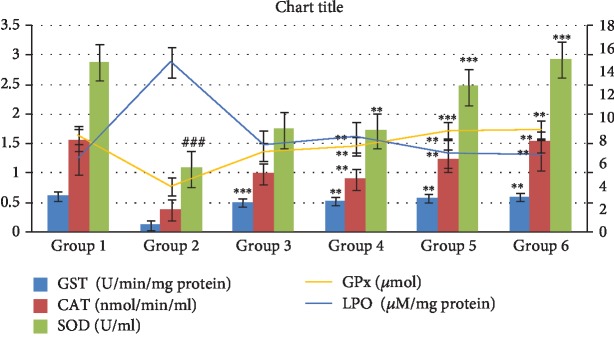
Effect *Catharanthus roseus* and ursolic acid and combination on body weight. ^∗∗^*P* < 0.01 is considered significant, ^∗∗∗^*P* < 0.001 is considered highly significant. Group 2 compared with Group 1, and comparison is considered significant. ^##^*P* < 0.01 is considered very significant; ^###^*P* < 0.001 is considered highly significant.

The effect of the treatment on body weight in STZ-induced diabetic rats is represented by the graph in Figure 1. It can be clearly seen that the body weight of Group 2 at the end of the study was significantly decreased (*P* < 0.01) by 32% (136.86 g), whereas Group 1 had almost no change. On the other hand, between the groups receiving single drug treatment, Group 4 (179.64 g) showed better recuperation of body weight than Group 3 (174.56 g). Contrastingly, Group 5 had the best result (201.76 g) as it was administered the combinational therapy and the results were strikingly similar to that of the standard drug treatment Group 6. The body weight analysis outlines that the combinational treatment has promising results and hence potential antidiabetic activity may be evident.

### 3.2. Blood Glucose Level

STZ-induced diabetic rats exhibited an increase in the level of blood glucose (*P* < 0.05); the glucose level peaked more than 3 times than that of Group 1 rats. Over the course of 4 weeks, on the treatment with single drugs, it was found that Group 3 and Group 4 treated with CREE and UA, respectively, had almost similar results where the blood glucose level lessened nearing the normal (Table 1). The decrease in the treatment group was significant as compared with Group 1 (*P* < 0.05 in all cases) and Group 2 (*P* < 0.05 in all cases). The combination treatment of rats in Group 5 with CREE and UA showed striking results as the blood glucose level (98.35 ± 2.45 mg/dl) in this group was below the normal rats (101.64 ± 2.82 mg/dl) at the end of the study. The result of Group 6 was considered the standard and hence it showed the best results in lowering the blood glucose level in rats (Table 1).

### 3.3. Plasma Insulin Level

Post STZ administration, on Day 0, the level of plasma insulin was found to be decreased significantly (*P* < 0.05) in the rats of all groups (below 7.00 *μ*U/ml) except Group 1 (15.65 ± 0.24 *μ*U/ml) as evident from Table 2. At the end of the study, it was observed that the levels of plasma insulin were successfully restored in animals of group 3, 4, 5, and 6 significantly (*P* < 0.01 in all cases). Group 5 which received the combined treatment of CREE and UA showed best restoration of the plasma insulin (13.65 ± 0.10 *μ*U/ml) in a significant manner.

### 3.4. Liver Glycogen Level

**Figure 2:**
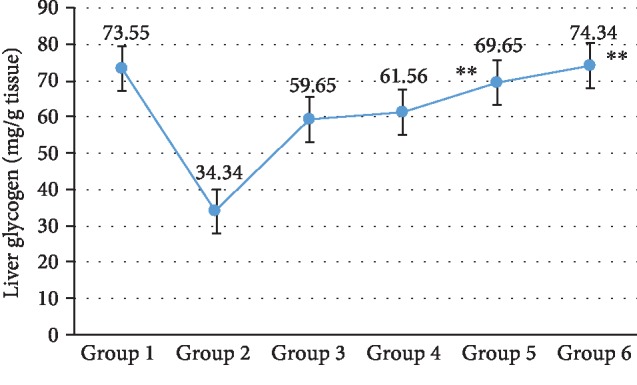
Effect *Catharanthus roseus* and ursolic acid and combination on liver glycogen level. ^∗∗^*P* < 0.01 is considered significant, ^∗∗∗^*P* < 0.001 is considered highly significant. Group 2 compared with Group 1, and comparison is considered significant. ^##^*P* < 0.01 is considered very significant; ^###^*P* < 0.001 is considered highly significant.

The effect of the treatments on liver glycogen storage level in STZ-induced diabetic rats is represented by the graph in Figure 2. Liver glycogen level was found to diminish in the rats administered with STZ, as in Group 2 it was found to decrease by more than 50% (34.34 mg/g tissue) as compared with Group 1 (73.55 mg/g tissue) (*P* < 0.01). On the administration of the combination of CREE and UA, the liver glycogen level was significantly restored (*P* < 0.01) in Group 5 (69.65 mg/g tissue) while the single treatment group receiving either CREE or UA showed noticeable elevation in the liver glycogen level (*P* < 0.01) in Group 3 and Group 4. The standard treatment Group 6 had almost normal level of liver glycogen.

### 3.5. Antioxidant Enzyme Level

**Figure 3:**
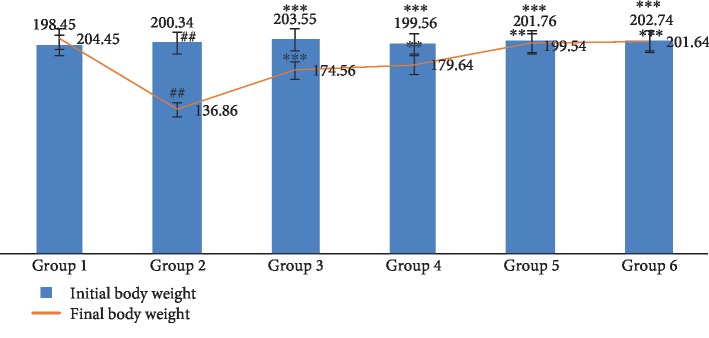
Effect *Catharanthus roseus* and ursolic acid and combination on antioxidant enzyme level. ^∗∗^*P* < 0.01 is considered significant; ^∗∗∗^*P* < 0.001 is considered highly significant. Group 2 compared with Group 1, and comparison is considered significant. ^##^*P* < 0.01 is considered very significant; ^###^*P* < 0.001 is considered highly significant.

The effect of CREE and UA on the activities of GST, SOD, CAT, GPx, and LPO in STZ-induced diabetic rats is illustrated in Figure 3. In the rats treated with STZ, the activities of GST, CAT, GPx, and SOD were noticeably reduced (*P* < 0.01), whereas LPO was elevated significantly (*P* < 0.01) as compared with the disease control Group 2 rats (see Figure 3). In posttreatment with CREE and/or UA, the results were reversed, and remarkable restoration of the antioxidant enzyme was observed at the end of the study. In line with our study, plants possessing anti-hyperglycemic effects are used as potential sources for drug development worldwide. Medicinal plants containing flavonoids, alkaloids, terpenoids, glycosides, etc. are often considered to possess anti-hyperglycemic activity.

## 4. Discussion

Our study was aimed at investigating the antidiabetic activity of the combination of CREE and UA in STZ-induced diabetes in rats and also the evaluation of antioxidant capacity of these herbal extracts in order to establish scientific evidence for the use of CREE and UA in the treatment of diabetes. In our study, we deduced that anti-hyperglycemic action of the combination of CREE and UA was mediated by their antioxidant properties.

*C. roseus* contain several biological substances like flavonoids, terpenoids, tannins, phenolics, and glycosides [12, 21]. Type 2 diabetes mellitus is resultant of structural and functional changes in the beta cells of pancreas and is characterized by the reduced insulin secretion. The elevated blood glucose and other biochemical deviations in the serum level are caused due to the deficiency of insulin or due to insulin resistance in target organs (IDF). Out of the several models available for the induction of diabetes, we used STZ model as per the facility available in our research laboratory. STZ is a derivative of nitrosourea and has been used in mimicking diabetes in rodents. STZ enhances the production of free radicals and the free radical scavenger system is inhibited, which gives rise to oxidative stress and finally damages pancreatic beta cells. The selective destruction of beta cells leads to deficiency in the serum levels of insulin consequently leading to hypoglycemic condition [22, 23]. In our study, we used a single dose of 50 mg/kg body weight of STZ for the induction of diabetic condition, following which the fasting blood glucose level elevated rapidly confirming the induction of diabetes in the rats under the study and it was also in confirmation of the previously reported studies [24–26].

Hyperglycemia is characterized by signs of polydipsia, polyuria, and loss of body weight. The reduction in body weight is associated with proteolysis in the skeletal muscle and the degradation of fats induced by non-availability of energy *via* breakdown of carbohydrates [27]. In this study, we found that the loss in body weight was noticeable in Group 2 rats, inferring a progressive proteolysis induced by the derangement of carbohydrate metabolism. The administration of combination of CREE and UA recuperated the body weight in Group 5 nearing to the normal and in congruence to the standard treatment Group 6. However, the restoration of body weight in the single treatment Group 3 and 4 was not that significant (Figure 1). The restoration of body weight showed that the treatment possibly worked in preventing muscle wasting by controlling the glycemic status.

The decreased level of insulin in the diabetic animals was due to the destruction of the beta cells in pancreas [28, 29]. The treatment of the CREE and UA combination showed best efficacy in Group 5, which was in equivalence to the activity of the standard drug in Group 6 (Table 2). It is assumed that it might be due to the stimulation of beta cell to secrete insulin or it may also be possible due to the regeneration of beta cells of pancreas. Our results corroborate the previously reported research studies of similar kind [30–32].

The synthesis of hepatic glycogen content is decreased due to diabetic condition, and it may be due to the low level of insulin in the body which inactivates glycogen synthase mechanism [33]. The decrease in glycogen synthesis has been reported in several previously reported research articles [34]. The restoration of hepatic glycogen content in Group 5 indicates that the combination of CREE and UA stimulates the glycogen synthase enzyme by boosting the insulin production from beta cells (Figure 2).

Antioxidant enzymes like SOD, CAT, GSH, and GPx play an important character in the prevention of damage caused by oxidative stress in the cells [20, 35, 36]. SOD scavenges upon the radical of superoxide and changes it into hydrogen peroxide. On the other hand, CAT reduces hydrogen peroxide into water molecule and hence protects the tissues from reactive hydroxyl radicals. GSH is also involved in the same process [37]. When the level of SOD is increased unproportionally to GPx increase, there is an overload of peroxide that builds up inside the cell [38]. In diabetic condition, elevated blood glucose can inactivate these antioxidant enzymes and hence an oxidative stress occurs which subsequently causes lipid peroxidation [39]. The activities of SOD, CAT, GSH, and GPx was elevated and restored to normal while that of the LPO was reduced to normalize; this led to the attenuation of oxidative stress (Figure 3).

## 5. Conclusion

The present study concludes that CREE and UA showed potent hypoglycemic activity in STZ-induced diabetes in Wistar strain rats when compared with the normal rats. This combination treatment of CREE and UA is found and shown to enhance the efficacy and reduce the side effects. Significant improvement in body weight, levels of serum insulin, liver glycogen, and antioxidant enzymes activity were observed with best effect in the combined treatment group rather than the single drug treatment group. More detailed investigations are required to further strengthen these novel findings and further researches required in order to determine the specific mechanism of action of the antidiabetic effect.

## Figures and Tables

**Table 1:** Effect *Catharanthus roseus* and ursolic acid and combination on blood glucose level (mg/dl).

Groups	Days				
	0 day	7^th^ day	14^th^ day	21^st^ day	28^th^ day
Group 1	098.65 ± 2.17	099.98 ± 2.46	102.86 ± 2.46	99.84 ± 1.84	101.64 ± 2.82
Group 2	285.47 ± 4.68	293.45 ± 4.57^##^	302.45 ± 3.67^###^	315.86 ± 6.33^###^	321.75 ± 5.46^##^
Group 3	275.26 ± 3.96	223.64 ± 3.674^∗^	158.35±2.57^∗∗^	110.85±246^∗∗^	109.46±5.46^∗∗^
Group 4	288.68 ± 4.63	215.85±3.75^∗∗∗^	145.76±2.64^∗∗∗^	105.45 ± 3.75^∗^	106.67±2.67^∗∗^
Group 5	283.46 ± 5.86	195.61±2.87^∗∗^	161.41±2.98^∗∗∗^	098.56±1.82^∗∗^	098.35±2.45^∗∗∗^
Group 6	271.96 ± 4.46	175.45±2.46^∗∗∗^	120.24±1.86^∗∗^	080.34±1.56^∗∗^	077.45±1.46^∗∗^

Values are expressed as Mean ± SEM, (*N* = 6). ^#^Groups as compared with Group 1; ^∗^groups as compared with Group 2; ^∗^*P* < 0.05; ^∗∗^*P* < 0.01; ^∗∗∗^*P* < 0.001.

**Table 2:** Effect *Catharanthus roseus* and ursolic acid and combination on plasma insulin level (*μ*U/ml).

Groups	Days				
	0 day	7^th^ day	14^th^ day	21^st^ day	28^th^ day
Group 1	15.65 ± 0.24	15.91 ± 0.11	15.34 ± 2.46	15.98 ± 0.12	15.78 ± 0.18
Group 2	06.94 ± 0.15	06.85 ± 4.57^##^	06.43 ± 0.17^#^	06.05 ± 0.16^##^	05.93 ± 0.31^##^
Group 3	06.86 ± 0.09	08.74 ± 3.674^∗^	09.62±0.14^∗∗^	10.33±0.21^∗∗∗^	10.41±0.20^∗∗^
Group 4	06.65 ± 0.25	08.56 ± 3.75^∗^	09.70±0.19^∗∗∗^	11.11 ± 0.12^∗^	11.46±0.21^∗∗^
Group 5	06.76 ± 0.16	09.57±2.87^∗∗∗^	10.46±2.98^∗∗∗^	13.34±0.21^∗∗∗^	13.65±0.10^∗∗^
Group 6	06.73 ± 0.14	10.11±0.14^∗∗^	12.56±0.16^∗∗^	16.01±0.09^∗∗^	15.97±0.18^∗∗∗^

Values are expressed as Mean ± SEM, (*N* = 6). ^#^Groups as compared with Group 1; ^∗^groups as compared with Group 2; ^∗^*P* < 0.05; ^∗∗^*P* < 0.01; ^∗∗∗^*P* < 0.001.

## Data Availability

We, the authors, support and endorse the FAIR Guiding Principles for scientific data management and stewardship-findability, accessibility, interoperability, and reusability. The experimental data used to support the findings of this study are included within the manuscript. However, additional information from this study may be obtained upon request to the corresponding author (aftab786sa@hotmail.com, or abdulsalam@kau.edu.sa). We ensured that Hindawi (Oxidative Medicine and Cellular Longevity) has the rights necessary for the proper administration of electronic rights and online dissemination of the manuscript entitled “Catharantus roseus combined with ursolic acid attenuates streptozotocin induced diabetes thourough insulin secretion and glycogen storage”.
